# The Urgency of Legal Issues Among Older Adults in the Context of the Fragmentation of Their “Good Objects”

**DOI:** 10.1192/j.eurpsy.2025.1586

**Published:** 2025-08-26

**Authors:** S. Georgakis, N. Zagorianakou, M. Gouva

**Affiliations:** 1 Research Laboratory of Patient, Family & Health Professional Psychology, Department of Nursing, School of Health Sciences, University of Ioannina, Ioannina, Greece

## Abstract

**Introduction:**

Conflicts concerning land ownership and inheritance disputes among older adults are prevalent and often lead to extreme and criminal behaviors. Even seemingly insignificant differences, such as disputes over a few square meters of land or a single tree, can escalate into violence or homicide.

**Objectives:**

This study aims to examine these phenomena through the lens of the fragmentation of the “good object,” as well as the accompanying sense of urgency experienced by older adults. This fragmentation refers to the loss of emotional and psychological foundations that provide a sense of security and identity. The study explores how these seemingly minor disputes, when they threaten the “good objects” of older adults, combined with their intense urgency to resolve these issues before it is too late, can turn into serious identity crises, potentially leading to extreme behaviors.

**Methods:**

Through the analysis of case studies and a psychoanalytic approach, the psychosocial factors influencing older adults’ decisions regarding the management of their property and their emotional connections to these assets are examined.

**Results:**

The findings reveal several key factors contributing to these conflicts, including: a) Emotional attachment to property stemming from years of hard work, b) Inheritance tensions and a sense of injustice, c) Financial insecurity, which makes property the most valuable asset for survival, d) Psychological pressure and isolation, which may lead to unpredictable behaviors, e) Lack of emotional control and traditional perceptions of honor and dignity, f ) A sense of loss of control as they age and the need to defend what they deem important.

**Conclusions:**

The psychoanalytic interpretation of these phenomena reveals that the fragmentation of the good object, which may be land or other property, is closely tied to the identity and mental well-being of older adults. These individuals experience an intense sense of urgency, as delays in resolving their legal issues can mean the loss of their identity. This urgent need to protect their “good objects” often leads to reactions, frequently violent, stemming from despair and the perception that their social fabric is abandoning them. For older adults, legal and property disputes are not merely material concerns but represent their last struggle to maintain their identity. It is essential to recognize the importance of these issues and provide them with the value they deserve.

**Disclosure of Interest:**

None Declared

